# Working from home during the COVID-19 pandemic and its longitudinal association with physical activity and sedentary behavior

**DOI:** 10.5271/sjweh.4027

**Published:** 2022-06-30

**Authors:** Bette Loef, Sandra H van Oostrom, Maaike van der Noordt, Lifelines Corona Research initiative, Karin I Proper

**Affiliations:** 1 Center for Nutrition, Prevention and Health Services, National Institute for Public Health and the Environment, Bilthoven, The Netherlands; 2 Center for Health and Society, National Institute for Public Health and the Environment, Bilthoven, The Netherlands; 3 Department of Public and Occupational Health, Amsterdam UMC, Vrije Universiteit Amsterdam, Amsterdam Public Health research institute, Amsterdam, The Netherlands

**Keywords:** home worker, hybrid worker, location worker, longitudinal study, physical inactivity, sitting

## Abstract

**Objective:**

Working from home during the COVID-19 pandemic has affected many workers’ daily life and possibly their physical activity behavior. We studied the longitudinal association of working from home during the pandemic with physical activity and sedentary behavior.

**Methods:**

Longitudinal data from 17 questionnaire rounds of the Lifelines COVID-19 cohort (March 2020–February 2021) were used. In total, 33 325 workers were included. In every round, participants reported their current work situation: location, home, or hybrid (working on location and from home). Physical activity levels and sedentary behavior before and during the pandemic were asked. Logistic generalized estimating equations adjusted for demographic/work/health covariates were used to study the association of work situation with physical activity and sedentary behavior.

**Results:**

Home workers were less likely to meet the recommended ≥150 minutes/week of moderate-to-vigorous-intensity activity during the pandemic than location workers [odds ratio (OR) 0.93, 95% confidence interval (CI) 0.90–0.96] and more likely to be less physically active than before the pandemic (OR 1.09, 95% CI 1.04–1.14). Furthermore, compared to location workers, home and hybrid workers were more likely to be more sedentary (sitting ≥8 hours/day) on workdays during than before the pandemic (OR 1.51, 95% CI 1.39–1.64/1.36–1.68, respectively).

**Conclusions:**

Compared to location workers, home workers (and to a lesser extent hybrid workers) were more often physically inactive and sedentary during than before the COVID-19 pandemic. As a substantial part of the working population may continue to work (partly) from home after the pandemic, workers should be supported to increase activity and reduce sitting while working from home.

In 2020, the entire world was affected by the COVID-19 pandemic (1). Besides the enormous impact of the SARS-CoV-2 virus on the health of millions of people, the measures taken to combat the virus also greatly influenced the lives and wellbeing of many people. One of the impactful containment measures that was taken on a large scale was the work-from-home mandate. From the start of the COVID-19 pandemic, the Dutch and many other governments asked workers to work from home unless this was impossible. For workers who used to work on location and suddenly had to work partly or fully from home, a working day and working conditions changed completely. Workers who used to commute to work, worked in an office setting, and interacted with colleagues face-to-face, now spent their working time at home. The change in workplace from working at location to the home setting thus changed workers’ daily routine, which may subsequently have altered their lifestyle behaviors, such as physical activity.

The beneficial effects of physical activity on preventing numerous chronic diseases and improving quality of life are well known (2, 3). However, approximately one in four adults worldwide did not meet the global physical activity recommendation of performing at least 150 minutes of moderate-intensity activity per week before the COVID-19 pandemic (2, 3). In The Netherlands, 45% of the adult population and 41% of the working adult population did not meet this recommendation in 2019 (4). The COVID-19 pandemic and subsequent measures to prevent the virus from spreading (eg, staying at home as much as possible, social distancing, closure of gyms and public parks) are likely to have limited opportunities to be physically active and thereby further reduced physical activity levels (5, 6).

While the evidence for a decrease in physical activity during the COVID-19 pandemic is growing (5–9), comprehensive insight on the role of working from home in this context is still lacking. A few studies have hypothesized or presented (preliminary) results indicating that working from home is associated with less physical activity and more sedentary behavior (10–14). In addition, Xiao et al (15) reported that less exercise was associated with a decreased overall physical and mental wellbeing among those working from home early in the pandemic. However, most of the studies so far have used cross-sectional data or relatively short follow-up periods. Due to the novelty of this global pandemic, more longitudinal research is needed to comprehensively study whether working from home has resulted in more physical inactivity and sedentary behavior, ideally with multiple measurements throughout the pandemic. As working from home is likely to remain partly in place after the pandemic for at least part of the workforce, such insights are needed to provide recommendations for workers, employers, and policy-makers to encourage healthy working from home practices in the future.

Therefore, the aim of the current study is to study the association between working from home (either fully or partly) and physical activity and sedentary behavior in a large population of Dutch workers, using data collected from March 2020 (start of the COVID-19 pandemic in The Netherlands) until February 2021.

## Methods

### Study design and population

In this prospective study, data were used from the Lifelines COVID-19 cohort, a cohort with the aim of studying the psychological and societal impact of the COVID-19 pandemic and potential risk factors of COVID-19 among the general Dutch population (16). The Lifelines COVID-19 cohort was initiated in March 2020, one month after the first COVID-19 case occurred in The Netherlands. Participants of the Lifelines COVID-19 cohort received (bi)weekly questionnaires on their work situation, lifestyle, health, and experiences during the pandemic from March 2020–July 2020, and monthly questionnaires from July 2020 onwards.

The Lifelines COVID-19 cohort is part of the larger Lifelines population cohort (17). This is a multi-­disciplinary prospective population-based cohort study examining in a unique three-generation design the health and health-related behaviors of 167 729 persons living in the north of The Netherlands. It employs a broad range of investigative procedures in assessing the biomedical, socio-demographic, behavioral, physical and psychological factors which contribute to the health and disease of the general population, with a special focus on multi-morbidity and complex genetics. All active adult participants from the Lifelines population cohort were invited to participate in the Lifelines COVID-19 cohort.

The current study comprises 17 questionnaire rounds of the Lifelines COVID-19 cohort, that were conducted between March 2020–February 2021 (supplementary material, www.sjweh.fi/article/4027, table S1). Working participants aged 18–67 years who completed at least one questionnaire round and who had data available on work situation, physical activity, and covariates were included in the analysis.

### Measures

*Work situation*. In each of the 17 questionnaire rounds, participants were asked what they currently did in their daily life (student; work; on disability; unemployed; retired; maternity leave; other). Participants who answered “I work” were asked to indicate their current work situation from one or more of the following responses: I work from home; I am laid off but am still being paid; I am laid off and am no longer being paid; I continue to work at the usual location (eg, office, factory, construction site); I continue to work at multiple sites for my job; I am forced to take sick leave or vacation time; other. For every round, participants who indicated to work at the usual location and/or at multiple sites for their job were labeled location workers, those who indicated to work from home were labeled home workers, and those who indicated to work at location as well as from home were labeled hybrid workers. Participants who solely chose one of the other options were not included in the analysis for that particular round. In addition, participants who were not working, being laid off, and/or taking sick leave or vacation time for the majority of the follow-up measurements were excluded from all analyses. For this purpose, two additional inclusion criteria were formulated: (i) participants were only included if they worked >75% of the rounds in which they participated, and (ii) if they, of those rounds in which they worked, worked >75% of the time on location and/or from home.

Besides the time-dependent work situation variable that was subject to change based on the input of every subsequent round and that was used in the longitudinal analysis, we also constructed an overall/fixed variable for work situation based on the work situation in the full year of follow-up (March 2020–February 2021). In this overall variable, participants were labeled (i) location workers if they worked on location and did not work from home in the entire year, (ii) home workers if they worked from home and did not work on location in the entire year, and (iii) hybrid workers if they worked both on location and from home in the entire year (but this did not necessarily had to be at the same time/questionnaire round, which is the case with the time-dependent work situation variable). The overall work situation variable was used to describe numbers and characteristics of location, home, and hybrid workers in the flowchart and descriptive information table.

*Physical activity*. In the Lifelines COVID-19 cohort, participants were asked three questions about moderate- and vigorous-intensity physical activity based on the Dutch Physical Activity Guidelines 2017 (18). Participants were asked “how many minutes of (moderately) intense activity did you do (eg, walking, biking or running)” in the last 7 days (rounds 1–6) or 14 days (rounds 7–17). In rounds 1 and 2, they were also asked how many minutes of (moderately) intense activity they performed each week before the COVID-19 pandemic. Responses could be one of five categories (<50; 50–100; 100–150; 150–180; >180 minutes in the last 7 days or <100; 100–200; 200–300; 300–360; >360 minutes in the last 14 days). Based on the global physical activity recommendation (3), answers were dichotomized into performing ≥150 minutes versus <150 minutes of at least moderate-intensity activity per week. Subsequently, for every available round, the following three dichotomous outcome measures were defined: (i) current level of moderate-to-vigorous-intensity activity during the pandemic (≥150 minutes versus <150 minutes activity per week); (ii) performing more moderate-to-vigorous-intensity activity during than before the pandemic (more activity (ie, shifting from performing <150 minutes to ≥150 minutes activity per week) versus similar/less activity); (iii) performing less moderate-to-vigorous-intensity activity during than before the pandemic (less activity (ie, shifting from performing ≥150 minutes to <150 minutes activity per week) versus similar/more activity).

Specifically for vigorous-intensity activity, participants were asked to answer the following statement “I do muscle and bone strengthening exercises, such as nordic walking, jumping rope, or weight training …” with more than; just as much; or less than in the period before the COVID-19 pandemic. Based on this information, two outcome measures were defined: (iv) performing more vigorous-intensity activity during than before the pandemic (more activity versus similar/less activity); (v) performing less vigorous-intensity activity during than before the pandemic (less activity versus similar/more activity).

*Sedentary behavior*. In round 6, participants were asked how much time they spent sitting on average per day (<1; 1; 2; 3; 4; 5; 6; 7; 8; 9; 10; 11; 12; >12 hours) in the past 7 days and before the COVID-19 pandemic. This question was based on the International Physical Activity Questionnaire Short Form (IPAQ-SF) (19). Questions were asked for work- and weekend days separately. In rounds 11 and 14–17, they were asked about time spent sitting per day in the past 14 days. Based on the distribution of sitting time in the study population and cut-offs for adverse health effects of sedentary behavior observed in previous work (20, 21), answers were dichotomized into sitting ≥8 versus <8 hours per day. Subsequently, the following outcome measures were defined (separately for work- and weekend days): (vi) current sedentary behavior during the pandemic (sitting ≥8 hours/day versus <8 hours/day); (vii) more sedentary during than before the pandemic (more sitting versus similar/less sitting); (viii) less sedentary during than before the pandemic (less sitting versus similar/more sitting).

### Covariates

Covariates from three different domains were included, namely demographic (age, sex, educational level, country of birth, household composition), work (occupation, occupational class, employment contract), and health (general health, testing positive for COVID-19) variables. Data on age, sex, education, country of birth, occupation, and occupational class were obtained from the Lifelines population cohort. Information on household composition (rounds 1–17), employment contract (rounds 1–10, 13, 16, 17), general health (rounds 1–2), and testing positive for COVID-19 (rounds 1–17) was obtained from one or more questionnaire rounds of the Lifelines COVID-19 cohort. In supplementary text S1, a complete description of the included covariates is presented.

### Statistical analysis

To gain insight into differences in the characteristics of the study population by overall work situation (location/home/hybrid workers) during the COVID-19 pandemic, the independent-samples t-test and the chi-square test were used. To visualize changes in work situation over time during the COVID-19 pandemic (time-dependent work situation variable), a graph was constructed representing the distribution of location, home, and hybrid workers per questionnaire round.

Logistic generalized estimating equations (GEE) analysis with an exchangeable correlation structure was used to study the longitudinal association between work situation and physical activity, and between work situation and sedentary behavior. Within GEE analysis, an adjustment is made for the dependency of multiple observations within an individual over time by modeling the within subject correlation matrix. GEE analysis instead of mixed model analysis was used because logistic GEE analysis has been found to be preferable in the estimation of regression coefficients when there is a dichotomous outcome variable (22). In the GEE analysis, both work situation and the outcome measures could vary over time. Location workers were used as the reference group. For all outcome measures, analyses were a priori adjusted for age, sex, educational level, country of birth, household composition, occupation, occupational class, employment contract, general health, and testing positive for COVID-19. The models with the outcome measures current moderate-to-vigorous-intensity activity and current sedentary behavior were additionally adjusted for moderate-to-vigorous-intensity activity/sedentary behavior before the COVID-19 pandemic.

P-values <0.05 were considered statistically significant. Analyses were conducted using IBM SPSS Statistics, version 25.0 (IBM Corporation, Armonk, NY, USA).

## Results

### Study population

In total, 140 145 active adult participants from the Lifelines population cohort were invited to participate in the Lifelines COVID-19 cohort (supplementary figure S1). Of these, 76 421 participants completed at least one questionnaire round in the period March 2020–February 2021. After excluding participants aged >67 years, those who were not employed >75% of their follow-up time, and those not working >75% of their time employed on location and/or from home, 43 116 participants remained in the study population. Next, 33 325 participants with information on past and current physical activity and covariates were included for the analyses with physical activity as outcome measure. Of these, 18 379 participants with information on past and current sedentary behavior were also included for the analyses with sedentary behavior as outcome measure (supplementary figure S1).

Home and hybrid workers were somewhat younger than location workers (48.5 and 48.8 years versus 50.4 years, table 1) (for an overview of all characteristics see supplementary table S2). The largest difference in demographic variables by work situation was found in educational level: 66.9% of home workers and 64.7% of hybrid workers had a high educational level compared to 23.7% of location workers. This difference also appeared in the observation that almost three quarters of home and hybrid workers were high-skilled white-collar workers, while this was the case for 42.3% of location workers. Home and hybrid workers reported to have a fair/poor health somewhat more often than location workers (4.8% and 4.1% versus 3.6%). However, homeworkers tested positive for COVID-19 less often during the study period than location workers (1.1% versus 2.6%).

**Table 1:** Characteristics of the study population stratified for location workers, home workers, and hybrid workers (who worked both on location and from home) during the COVID-19 pandemic from March 2020–February 2021 (N=33 325). [SD=standard deviation.]

	Location workers (N=16 043)			Home workers (N=8473)			Hybrid workers (N=8809)		
		
	Mean (SD)	%	N	Mean (SD)	%	N	Mean (SD)	%	N
Age (in years)	50.4 (8.9)			48.5 (9.3) ^a^			48.8 (9.5) ^b^		
Sex (female)		59.9	9614		55.7 ^a^	4719		60.8	5360
Educational level									
Low		20.9	3354		4.8 ^a^	408		5.0 ^b^	437
Middle		55.4	8892		28.3 ^a^	2400		30.3 ^b^	2673
High		23.7	3797		66.9 ^a^	5665		64.7 ^b^	5699
Household composition									
Living alone		7.7	1234		7.8	662		6.9 ^b^	610
Living together with children		1.5	246		2.6 ^a^	222		2.0 ^b^	178
Living together with adults		52.2	8376		44.8 ^a^	3800		46.9 ^b^	4134
Living together with children and adults		37.1	5948		43.6 ^a^	3693		42.7 ^b^	3764
Living together but unknown with whom		1.5	239		1.1 ^a^	96		1.4	123
Occupation									
High-skilled white-collar		42.3	6792		73.8 ^a^	6257		72.2 ^b^	6360
Low-skilled white-collar		33.6	5398		22.3 ^a^	1889		22.5 ^b^	1981
High-skilled blue-collar		11.9	1909		2.3 ^a^	199		3.6 ^b^	317
Low-skilled blue-collar		12.1	1944		1.5 ^a^	128		1.7 ^b^	151
General health (fair/poor)		3.6	581		4.8 ^a^	406		4.1 ^b^	363
Testing positive for COVID-19 (yes)		2.6	416		1.1 ^a^	97		2.8	251
≥150 minutes of moderate-to-vigorous-intensity activity per week before COVID-19 pandemic (yes)		42.0	6744		44.3 ^a^	3753		41.8	3683
Sitting ≥8 hours per workday before COVID-19 pandemic (yes)		15.9	1349		51.3 ^a^	2009		36.7 ^b^	2193
Sitting ≥8 hours per weekend day before COVID-19 pandemic (yes)		9.0	765		14.0 ^a^	548		12.2 ^b^	729

a Statistically significant difference (P<0.05) between homeworkers and location workers tested with independent-samples t-test and chi-square test.

b Statistically significant difference (P<0.05) between hybrid workers and location workers tested with independent-samples t-test and chi-square test.

Table 1 also shows that home workers more often performed ≥150 minutes of moderate-to-vigorous-intensity activity per week before the pandemic than location workers (44.3% versus 42.0%). Furthermore, home and hybrid workers were more sedentary on workdays (51.3% and 36.7% versus 15.9% sitting ≥8 hours/day) and weekend days (14.0% and 12.2% versus 9.0% sitting ≥8 hours/day) than location workers before the pandemic.

### Working from home during the COVID-19 pandemic

**Figure 1:**
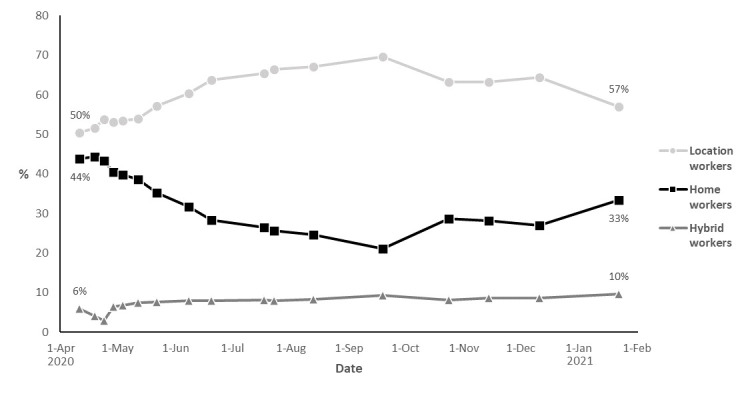
Percentages of location workers, home workers, and hybrid workers at the 17 different questionnaire rounds during the study period (March 2020–February 2021) among 33 325 workers. Every datapoint represents the median date of the particular questionnaire round. NB: not all 33 325 workers participated in every questionnaire round, percentages are based on the following numbers of included workers per round: 1: N=24 702; 2: N=24 060; 3: N=21 644; 4: N=21 995; 5: N=20563; 6: N=18 940; 7: N=18 003; 8: N=15 782; 9: N=14996; 10: N=13203; 11: N=14 375; 12: N=15 108; 13: N=15 171; 14: N=14 447; 15: N=14 452; 16: N=13 297; 17: N=13 939.

Figure 1 shows that during the first questionnaire round (March–April 2020) of the Lifelines COVID-19 cohort, 44% of the participants worked exclusively from home. From May to September, the proportion of home workers decreased, with 21% working from home in round 13 (September 2020). In September, the number of confirmed COVID-19 cases strongly increased in The Netherlands, and new containment measures were announced. This is possibly reflected by the increase in the proportion of home workers in the period September–November 2020. Starting from December 15^th^ until after the end of the study period, The Netherlands was in lockdown and the percentage of participants who worked exclusively from home increased to 33% in January and February 2021.

### Working from home and physical activity

**Figure 2:**
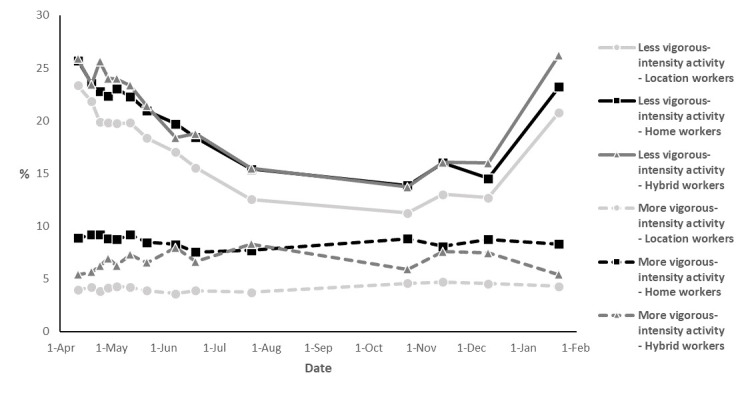
Percentages of location workers, home workers, and hybrid workers who reported to do more (dashed lines) and less (solid lines) vigorous-intensity activity during the COVID-19 pandemic than before the pandemic. Every datapoint represents the median date of the particular questionnaire round.

After adjustment for all covariates, home workers were less likely to perform ≥150 minutes/week of moderate-to-vigorous-intensity activity during the pandemic than location workers [odds ratio (OR) 0.93, 95% confidence interval (CI) 0.90–0.96] (table 2). Compared to location workers, home workers were also less likely to be more physically active during the pandemic than before the pandemic (OR 0.92, 95% CI 0.89–0.96), and more likely to be less physically active than before the pandemic (OR 1.09, 95% CI 1.04–1.14). Thus, home workers had a 1.09 times higher odds than location workers to shift from adhering to global physical activity recommendation before the pandemic to not adhering to this guideline during the pandemic. For moderate-to-vigorous-intensity activity, no differences were observed between hybrid and location workers. However, for vigorous-intensity activity, both home (OR 1.69, 95% CI 1.58–1.80) and hybrid (OR 1.32, 95% CI 1.20–1.44) workers were more likely to be more physically active than before the pandemic compared to location workers. Interestingly, home (OR 1.36, 95% CI 1.30–1.41) and hybrid (OR 1.18, 95% CI 1.13–1.24) workers were also more likely to be less vigorously active than before the pandemic compared to location workers. This finding can be explained by the fact that the vigorous-intensity activity level of location workers largely remained unchanged during compared to before the pandemic, while home and hybrid workers relatively often started to perform more or less vigorous-intensity activity during the pandemic. This observation is illustrated in figure 2, which also shows that most workers (independent of work situation) performed similar levels of vigorous-intensity activity during as before the pandemic. Between 11% and 26% of workers reported doing less vigorous-intensity activity somewhere during the pandemic, and between 4% and 9% reported more vigorous-intensity activity.

**Table 2:** Effect estimates ^a^ of the longitudinal associations between work situation and physical activity (N=33 325). Reference group=location workers. [CI=confidence interval; OR=odds ratio].

Physical activity outcome measures	Home workers		Hybrid workers	
	
	OR	95% CI	OR	95% CI
Current moderate-to-vigorous-intensity activity during pandemic (≥150 minutes vs. <150 minutes per week)	0.93 ^b^	0.90–0.96	1.02	0.98–1.07
More moderate-to-vigorous-intensity activity than before pandemic (more activity vs. similar/less activity)	0.92 ^b^	0.89–0.96	1.04	0.99–1.10
Less moderate-to-vigorous-intensity activity than before pandemic (less activity vs. similar/more activity)	1.09 ^b^	1.04–1.14	1.04	0.99–1.11
More vigorous-intensity activity than before pandemic (more activity vs. similar/less activity)	1.69 ^b^	1.58–1.80	1.32 ^b^	1.20–1.44
Less vigorous-intensity activity than before pandemic (less activity vs. similar/more activity)	1.36 ^b^	1.30–1.41	1.18 ^b^	1.13–1.24

a Adjusted for age, sex, educational level, country of birth, household composition, occupation, occupational class, employment contract, general health, testing positive for COVID-19. The fully adjusted model for the outcome measure current moderate-to-vigorous-intensity activity during the pandemic is additionally adjusted for moderate-to-vigorous-intensity activity before the pandemic. In total, 251 195 observations on physical activity during 14 questionnaire rounds were available for 33 325 participants.

b P<0.05.

### Working from home and sedentary behavior

Home and hybrid workers had 1.94 (95% CI 1.83–2.06) and 1.73 (95% CI 1.59–1.88) times more odds, respectively, to sit ≥8 hours on workdays during the pandemic than location workers after adjustment for covariates (table 3). Home (OR 1.51, 95% CI 1.39–1.64) and hybrid (OR 1.51, 95% CI 1.36–1.68) workers were also more likely to be more sedentary on workdays during than before the pandemic compared to location workers. For these sedentary behavior outcome measures on weekend days, associations in the same direction were observed by work situation, but with smaller effect estimates. In addition, compared to location workers, home workers were less likely to be less sedentary on weekend days during than before the pandemic (OR 0.72, 95% CI 0.64–0.81). In supplementary tables S3 and S4, the crude and the different adjustment models for demographic, work, and health variables of the longitudinal associations between work situation and physical activity (table S3) and sedentary behavior (table S4) are presented.

**Table 3:** Effect estimates ^a^ of the longitudinal associations between work situation and sedentary behavior (N=18 379). Reference group=location workers. [CI=confidence interval; OR=odds ratio].

Sedentary behavior outcome measures	Home workers		Hybrid workers	
	
	OR	95% CI	OR	95% CI
Current sedentary behavior on workdays during the pandemic (sitting ≥8 hours vs. <8 hours per day)	1.94 ^b^	1.83–2.06	1.73 ^b^	1.59–1.88
More sedentary on workdays during than before the pandemic (more sitting vs. similar/less sitting)	1.51 ^b^	1.39–1.64	1.51 ^b^	1.36–1.68
Less sedentary on workdays during than before the pandemic (less sitting vs. similar/more sitting)	0.95	0.87–1.03	1.06	0.95–1.18
Current sedentary behavior on weekend days during the pandemic (sitting ≥8 hours vs. <8 hours per day)	1.32 ^b^	1.23–1.41	1.36 ^b^	1.23–1.50
More sedentary on weekend days during than before the pandemic (more sitting vs. similar/less sitting)	1.14 ^b^	1.05–1.24	1.36 ^b^	1.22–1.52
Less sedentary on weekend days during than before the pandemic (less sitting vs. similar/more sitting)	0.72 ^b^	0.64–0.81	1.01	0.88–1.16

a Adjusted for age, sex, educational level, country of birth, household composition, occupation, occupational class, employment contract, general health, testing positive for COVID-19. The fully adjusted model for the outcome measures current sedentary behavior during the pandemic are additionally adjusted for sedentary behavior before the pandemic. In total, 73 557 observations on sedentary behavior during 6 questionnaire rounds were available for 18 379 participants.

b P<0.05.

## Discussion

In this large longitudinal study, home workers during the COVID-19 pandemic (March 2020–January 2021) were less often moderately-to-vigorously active than before the pandemic compared to location workers. Compared to location workers, home and hybrid workers were also more often sedentary during than before the pandemic. For vigorous-intensity activity, two groups could be identified where, compared to location workers, part of the home and hybrid workers was more vigorously active while another part of the home and hybrid workers was less vigorously active during than before the pandemic.

Several previous studies have also reported an association of working from home during the COVID-19 pandemic with less moderate-to-vigorous-intensity activity (12–14) and more sedentary behavior (11–14). A recent systematic review on changes in workers’ physical activity and sedentary behavior during the COVID-19 pandemic concluded that work from home policies have impaired physical activity levels and increased sedentary behavior among workers (23). However, most of these previous studies compared physical activity levels of home workers during the beginning of the COVID-19 pandemic with physical activity levels before the pandemic, without using location workers as a reference group. A cross-sectional Japanese study did compare home with location workers, and found, similar to our findings, home workers to spend less time in moderate-to-vigorous-intensity activity and more time sedentary during working hours than those not working from home (12). To our knowledge, the current study is the first study with multiple measurements during almost one year of the COVID-19 pandemic to report a longitudinal association between working from home and reduced physical activity and increased sedentary behavior.

One possible explanation for home workers’ reduced physical activity levels and increased sedentary behavior compared to location workers is the decrease in active transportation to and from work among home workers. In 2019, >25% of commuting trips were done by bicycle in The Netherlands (24). Furthermore, a study conducted before the pandemic indicated that office workers take most steps on a workday during commuting hours (25). For many home workers, the loss of these modes of active transportation may have resulted in a substantial decrease in daily activity. Another explanation for our findings may be reduced activity at the workplace. Home workers may be sitting at their desk for most of their workday with more screen time than when working on location (26) and without having to walk to and from meetings. In general, home workers may be walking less on a working day due to the smaller size of their work area (12).

For vigorous-intensity activity, our results indicate a dichotomy within the group of home and hybrid workers during the pandemic, where some home and hybrid workers became more vigorously active, while others became less vigorously active than before the pandemic. In general, location workers were more likely to report just as much muscle and bone strengthening exercises during and before the pandemic, whereas changes in vigorous-intensity activity during the pandemic were more apparent among home and hybrid workers. Home workers who engaged in vigorous-intensity activity less often may have been less motivated to do so and/or may have perceived that there were less possibilities to exercise due to the advice to stay at home as much as possible, limited access to public places to be physically active (eg, public parks), closure of sports facilities, and additional responsibilities for school-aged children at home (5, 23). In contrast, other home workers may have been more motivated to exercise and may have found more opportunities to exercise during the working day (eg, during lunch time or between meetings) or used additional leisure-time during the pandemic to exercise before or after work (27). Prior research also indicates that some people have actually started to exercise more during the COVID-19 pandemic, for example by engaging in home training (27). However, more research is needed to investigate underlying reasons for exercising more or less during the COVID-19 pandemic, because it could offer starting points for interventions to stimulate vigorous-intensity activity among home workers. Nevertheless, as observed in the current study, an increase in vigorous-intensity activity among home workers may not necessarily lead to a decrease in sedentary time, because short bouts of intense activity (eg, weight training) do not make up a large part of the time and workers can still be sedentary for most of the day. Therefore, specific attention may be needed to reduce sedentary behavior among home workers.

At the beginning of the pandemic in March/April 2020, 44% of workers in the current study were fully working from home. This is comparable to another study among a representative group of Dutch citizens that reported 39% of workers to work (almost) completely from home in this period, while this was only 6% in 2019 (28). Besides the downsides of working from home, it also has advantages such as a reduction of commuting time and providing better opportunities to combine work and private life (29–32). Some studies have also reported increased productivity as a result of working from home (32, 33). Expectations are that many home workers will (partly) continue to work from home after the end of the COVID-19 pandemic (28, 30, 31). Part of these workers may consciously choose to alternate between working at their home office and on location. Additionally, some employers may also ask workers to do so or permit and facilitate their employees to work from home while this was not the case before the pandemic. This emphasizes the importance of the current findings and the importance of focusing on a healthy work environment at home that encourages sufficient physical activity and prevents too much sitting. A decrease in physical activity in a substantial part of the working population could lead to more health problems, both physically and mentally (15). Therefore, future research is needed to examine ways to create a supportive work environment at home (eg, active workstations, digital tools, rearrangement of lunch breaks (34–36), and addressing unfavorable working conditions at the home office (37) to reduce sitting and increase physical activity) and to develop informed guidelines for workers, employers, and policy makers to encourage physical activity at and around the home office.

### Strengths and limitations

A strength of the current study is its longitudinal design with multiple measurements of work situation, physical activity, and sedentary behavior over the course of almost one year of the COVID-19 pandemic. Other strengths are the large sample size and that analyses were adjusted for a wide range of covariates including demographic, work, and health variables. In addition, physical activity and sedentary behavior prior to the pandemic was also taken into account in the analyses.

Location workers and home workers differed substantially in baseline characteristics, such as occupational class and educational level. While analyses were adjusted for demographic, work, and health variables, residual confounding may therefore still exist. To study the association of working from home with physical activity and sedentary in a population where location workers and home workers were more alike, post-hoc analyses were conducted separately for white-collar workers (supplementary tables S5 and S6) and for workers with a high educational level (tables S7 and S8). The results of the post-hoc analyses among these specific groups of workers were virtually the same as the results for the total study population, which adds to the robustness of our findings.

In the current study, physical activity and sedentary behavior were based on self-report, which is less reliable than objective measures of these behaviors (38, 39). Therefore, recall bias, particularly in the questions about physical activity and sedentary behavior prior to the COVID-19 pandemic, cannot be ruled out. Nevertheless, self-reported measures are useful for comparing physical activity levels between groups, which was the purpose of the current study. In addition, the use of accelerometers would not have been feasible in the current study due to the large number of participants and multiple measurements. While the categorization of outcome measures into adhering to the global physical activity recommendation and more/less activity compared to before the pandemic provides insight into the clinical relevance of results, the categorization could lead to misclassification in these measures. However, since we have no reason to assume that this misclassification would be different depending on work situation, the impact of this bias may be limited.

Regarding the interpretation of the regression coefficients, it should be noted that regression coefficients estimated with GEE analysis combine the between-subject and within-subject effects. However, because the majority of workers did not change their work situation during the study period with 74% of workers being either stable location workers or stable home workers, the regression coefficients of the current study will largely reflect between-subject differences.

In this large longitudinal study, workers participated on average in 8 out of 14 questionnaire rounds on physical activity and 4 out of 6 questionnaire rounds on sedentary behavior. The large number of questionnaire rounds in a relatively short period of time in this study may have negatively influenced participation rates. Furthermore, being older, female, and having a higher educational level was associated with completing more questionnaire rounds. Therefore, bias due to selective inclusion might exist, which may affect the generalizability of our findings. As blue-collar workers were underrepresented in the study population, our results may apply mostly to white-collar workers.

Due to the unique changes in work situation during the COVID-19 pandemic, more longitudinal research with a long follow-up time is needed to confirm our findings. During the COVID-19 pandemic, workers and employers were unprepared and rushed to start working from home, which may have resulted in unfavorable work stations and a lack of policies to support healthy working environments. Therefore, insight is needed on whether similar associations between working from home and physical (in)activity as reported in the current study, can be expected after the COVID-19 pandemic.

### Concluding remarks

The COVID-19 pandemic has resulted in an enormous change in the work situation and working conditions of many workers worldwide. The current study shows that working from home during the COVID-19 pandemic was associated with reduced moderate-to-vigorous-intensity activity and increased sedentary behavior. Furthermore, an association of working from home with less as well as more vigorous-intensity activity was observed, suggesting large individual differences in how work situation may affect vigorous-intensity activities. These results imply that efforts are needed to support current and future home workers, employers, and policy makers in establishing healthy working from home practices to encourage workers to engage in sufficient physical activity and to reduce their sitting time in order to promote the health of home workers.

## Supplementary material

Supplementary material

## Protection of research participants

Approval of the Lifelines Cohort study was obtained from the Medical Ethics Committee of the University Medical Center Groningen, The Netherlands. Informed consent was obtained from all participants.
