# Supplementary material for: Working from home during the COVID-19 pandemic and its longitudinal association with physical activity and sedentary behavior
Source: Scand J Work Environ Health. 2022 Jun 30;48(5):380–90. doi: 10.5271/sjweh.4027 (PMC9527786; doi:10.5271/sjweh.4027)
Supplement: Supplementary material [file SJWEH-48-380-S001.pdf]

Working from home during the COVID-19 pandemic and its longitudinal association with physical activity and sedentary behavior[1]

by Bette Loef, PhD,[2] Sandra H van Oostrom, PhD, Maaike van der Noordt, PhD, Lifelines Corona Research initiative, Karin I Proper, PhD

1. SUPPLEMENTARY INFORMATION
2. Correspondence to: Bette Loef, PhD, Center for Nutrition, Prevention and Health Services, National Institute for Public Health and the Environment, P.O. Box 1, 3720 BA, Bilthoven, The Netherlands. [E-mail: bette.loef@rivm.nl]

Text S1. Covariates

Home workers and location workers are likely to differ with regard to their demographic characteristics and their jobs and corresponding work characteristics (Adams-Prassl A, Boneva T, Golin M, Rauh C. Work that can be done from home: Evidence on variation within and across occupations and industries. Labour Econ. 2022;74:102083). As these demographic and work characteristics could potentially be associated with physical activity and sedentary behavior levels, covariates from both these domains were included. Furthermore, baseline health differences and exposure to and infection with COVID-19 may also differ between home workers and location workers. Therefore, these covariates were also included. Data on the variables age, sex, education, country of birth, occupation, and occupational class were obtained from the Lifelines population cohort. Within the Lifelines population cohort, a baseline assessment (round 1A, 2007-2013), a second assessment (round 2A, 2014-2017), and a third assessment (round 3A, 2019-2023 (still ongoing)) have been conducted. These assessments consist of questionnaires, physical examinations, and collection of biosamples. In between these assessments, participants also received additional questionnaires in 2011-2014 (round 1B), 2012-2015 (round 1C), and 2016-2019 (round 2B). Information on the variables household composition (round 1-17), employment contract (round 1-10, 13, 16, 17), general health (round 1-2), and testing positive for COVID-19 (round 1-17) was obtained from one or more questionnaire rounds of the Lifelines COVID-19 cohort.

Demographic variables

Age was expressed as age in years at baseline, and sex was categorized into male or female. Participants were asked about their educational level in round 1A, 2A, 2B, and 3A of the Lifelines population cohort. Subsequently, a variable was created for the highest educational level (low; middle; high) reported. Country of birth (Netherlands; other country) was assessed in round 1A. Household composition (living alone; living together with adults; living together with children; living together with children and adults; living together but unknown with whom) was based on questions in round 1-17 of the Lifelines COVID-19 cohort.

Work variables

The work variables occupation (high-skilled white-collar; low-skilled white-collar; high-skilled blue-collar; low-skilled blue-collar) and occupational class (educational; creative and linguistic; commercial; business economics and administrative; managers; public

administration, security, and legal; technical; ICT; agricultural; care and welfare; service; transport and logistics; other occupations) were derived from the most recent available 2008 International Standard Classification of Occupations (ISCO) score from round 1A, 1B, 1C, 2A, and 2B of the Lifelines population cohort. The categorization of occupational class was based on the Dutch ROA-CBS 2014 occupational categorization system (BRC 2014) (Fouarge D, Dijksman S. Beroepenindeling ROA-CBS 2014 (BRC 2014). Maastricht: Maastricht University, Research Centre for Education and the Labour Market (ROA); 2015). In round 1-10, 13, 16, and 17 of the Lifelines COVID-19 cohort, participants were asked what kind of work contract they had (permanent; temporary; zero hour, flexible, on call; freelance; other). Next, participants were categorized into having exclusively a permanent contract, having both a permanent and non-permanent contract, and having exclusively a non-permanent contract during the study period.

Health variables

Baseline general health status in round 1 of the Lifelines COVID-19 cohort was used (excellent/very good/good; fair/poor), except if participants only reported their general health status in round 2, then this round was used. Lastly, for every questionnaire round, it was determined whether participants had tested positive for COVID-19 (yes; no) and this measure was subsequently included as a time-dependent covariate.

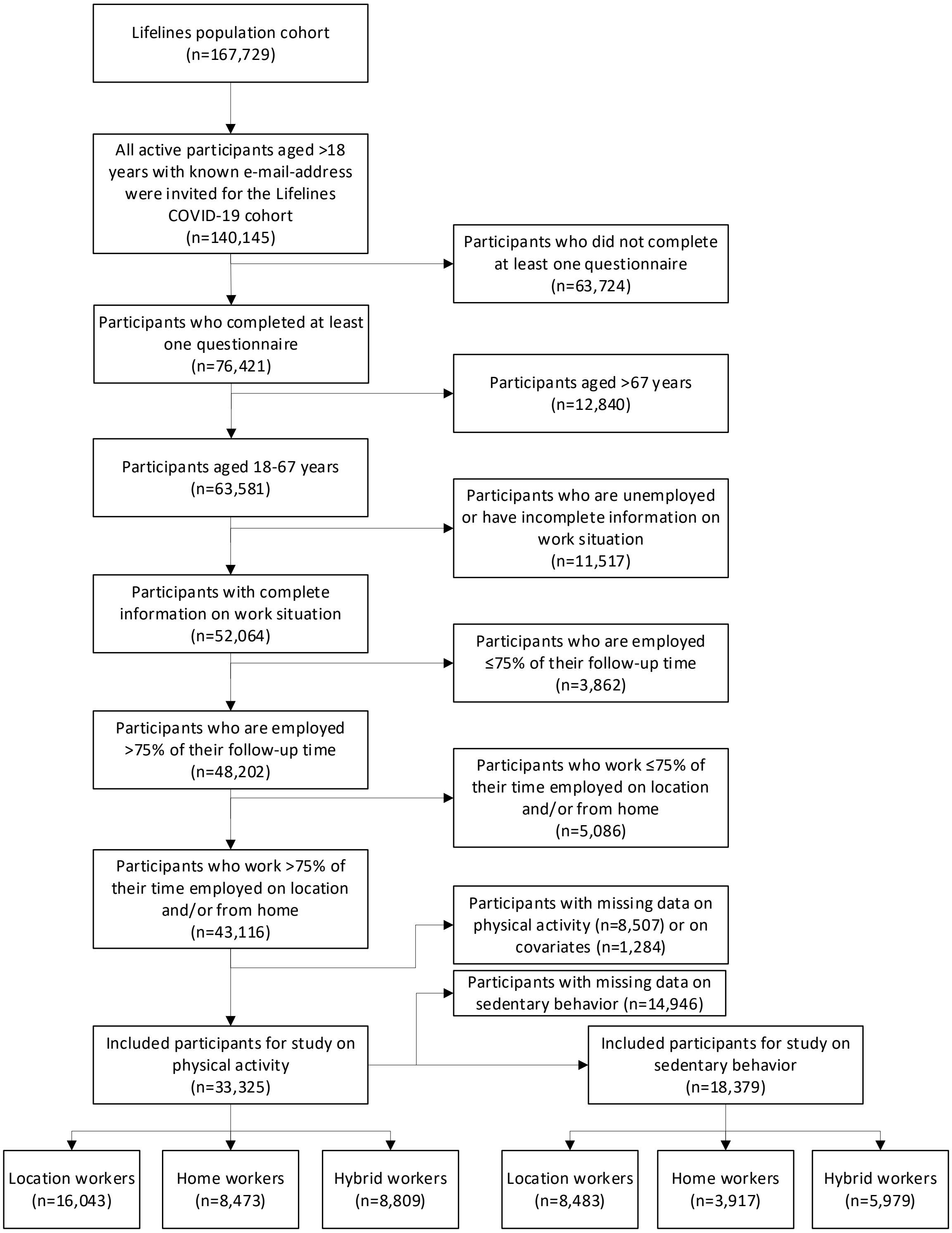

Figure S1. Flowchart of study participants.

Table S1. Overview of the questionnaire rounds of the Lifelines COVID-19 cohort from March 2020 to February 2021.

Round	Date	Work situation question	Physical activity (PA) questions	Sedentary behavior (SB) questions
1	Mar to Apr 2020	Current work situation	Current PA, past PA	-
2	Apr to May 2020	Current work situation	Current PA, past PA	-
3	Apr to May 2020	Current work situation	Current PA	-
4	Apr to May 2020	Current work situation	Current PA	-
5	Apr to May 2020	Current work situation	Current PA	-
6	Apr to May 2020	Current work situation	Current PA	Current SB, past SB
7	May 2020	Current work situation	Current PA	-
8	May to Jun 2020	Current work situation	Current PA	-
9	Jun 2020	Current work situation	Current PA	-
10	Jul 2020	Current work situation	-	-
11	Jul to Aug 2020	Current work situation	Current PA	Current SB
12	Jul to Sep 2020	Current work situation	-	-
13	Sep 2020	Current work situation	-	-
14	Oct to Nov 2020	Current work situation	Current PA	Current SB
15	Nov 2020	Current work situation	Current PA	Current SB
16	Dec 2020	Current work situation	Current PA	Current SB
17	Jan to Feb 2021	Current work situation	Current PA	Current SB

Table S2. Characteristics of the study population stratified for location workers, home workers, and hybrid workers (who worked both on location and from home) during the COVID-19 pandemic from March 2020-February 2021 (n=33,325). [SD=standard deviation]

	**Location workers (n=16,043)**		**Home workers (n=8,473)**		**Hybrid workers (n=8,809)**	
	Mean or %	SD or n	Mean or %	SD or n	Mean or %	SD or n
Demographic variables						
Age (in years)	50.4	8.9	48.5[a]	9.3	48.8[b]	9.5
Sex (% female)	59.9	9614	55.7[a]	4719	60.8	5360
Educational level (%)						
Low	20.9	3354	4.8[a]	408	5.0[b]	437
Middle	55.4	8892	28.3[a]	2400	30.3[b]	2673
High	23.7	3797	66.9[a]	5665	64.7[b]	5699
Household composition (%)						
Living alone	7.7	1234	7.8	662	6.9[b]	610
Living together with children	1.5	246	2.6[a]	222	2.0[b]	178
Living together with adults	52.2	8376	44.8[a]	3800	46.9[b]	4134
Living together with children and adults	37.1	5948	43.6[a]	3693	42.7[b]	3764
Living together but unknown with whom	1.5	239	1.1[a]	96	1.4	123
Born in the Netherlands (% no)	1.8	286	2.4[a]	201	1.8	158
Work variables						
Occupation (%)						
High-skilled white-collar	42.3	6792	73.8[a]	6257	72.2[b]	6360
Low-skilled white-collar	33.6	5398	22.3[a]	1889	22.5[b]	1981
High-skilled blue-collar	11.9	1909	2.3[a]	199	3.6[b]	317
Low-skilled blue-collar	12.1	1944	1.5[a]	128	1.7[b]	151
Occupational class (%)						
Educational occupations	2.5	403	9.6[a]	815	16.9[b]	1487
Creative and linguistic occupations	0.8	129	3.3[a]	277	2.3[b]	200
Commercial occupations	9.2	1469	8.0[a]	676	6.9[b]	609
Business economics and administrative occupations	15.8	2542	35.9[a]	3046	27.1[b]	2387
Managers	4.2	674	6.3[a]	531	6.6[b]	582
Public administration, security, and legal occupations	2.5	394	6.1[a]	517	4.4[b]	387
Technical occupations	16.4	2625	7.0[a]	597	8.5[b]	748
ICT occupations	1.0	168	10.8[a]	918	4.4[b]	390
Agricultural occupations	1.9	307	1.3[a]	107	1.7	148
Care and welfare occupations	32.3	5185	8.8[a]	745	17.9[b]	1580
Service occupations	8.0	1284	2.2[a]	189	2.5[b]	224
Transport and logistics occupations	5.1	821	0.6[a]	53	0.7[b]	63
Other	0.3	42	0.0[a]	2	0.0[b]	4
Employment contract (%)						
Permanent contract	79.8	12799	76.1[a]	6449	72.5[b]	6387
Permanent and non-permanent contract	6.1	977	4.8[a]	409	10.5[b]	929

Non-permanent contract	14.1	2267	19.1[a]	1615	16.9[b]	1493
Health variables						
General health (% fair/poor)	3.6	581	4.8[a]	406	4.1[b]	363
Testing positive for COVID-19 (% yes)	2.6	416	1.1[a]	97	2.8	251
Past physical activity & sedentary behavior						
≥150 minutes of moderate-to-vigorous-intensity activity per week before COVID-19 pandemic (% yes)	42.0	6744	44.3[a]	3753	41.8	3683
Sitting ≥8 hours per workday before COVID-19 pandemic (% yes)	15.9	1349	51.3[a]	2009	36.7[b]	2193
Sitting ≥8 hours per weekend day before COVID-19 pandemic (% yes)	9.0	765	14.0[a]	548	12.2[b]	729

[a] Statistically significant difference (p<0.05) between homeworkers and location workers tested with independent-samples t-test and chi-square test.

[b] Statistically significant difference (p<0.05) between hybrid workers and location workers tested with independent-samples t-test and chi-square test.

Table S3. Effect estimates of all adjustment models[a] of the longitudinal associations between work situation and physical activity (n=33,325). Reference group=location workers. [CI=confidence interval; OR=odds ratio].

		**Home workers**		**Hybrid workers**	
Physical activity outcome measures	*Model*	*OR*	*95% CI*	*OR*	*95% CI*
Current moderate-to-vigorous-intensity activity during pandemic (≥150 minutes vs. <150 minutes per week)	Model 1	1.01	0.98–1.04	1.05[b]	1.01–1.10
	Model 2	0.95[b]	0.92–0.98	1.02	0.98–1.06
	Model 3	0.93[b]	0.90–0.95	1.00	0.96–1.04
	Model 4	0.93[b]	0.90–0.96	1.00	0.96–1.05
	Model 5	0.93[b]	0.90–0.96	1.02	0.98–1.07
More moderate-to-vigorous-intensity activity than before pandemic (more activity vs. similar/less activity)	Model 1	0.98	0.95–1.02	1.09[b]	1.04–1.14
	Model 2	0.95[b]	0.91–0.98	1.06[b]	1.01–1.12
	Model 3	0.92[b]	0.89–0.96	1.04	0.99–1.10
	Model 4	0.92[b]	0.89–0.96	1.04	0.99–1.10
Less moderate-to-vigorous-intensity activity than before pandemic (less activity vs. similar/more activity)	Model 1	1.08[b]	1.04–1.13	1.04	0.99–1.10
	Model 2	1.08[b]	1.03–1.12	1.03	0.98–1.09
	Model 3	1.09[b]	1.04–1.14	1.04	0.98–1.10
	Model 4	1.09[b]	1.04–1.14	1.04	0.99–1.11
More vigorous-intensity activity than before pandemic (more activity vs. similar/less activity)	Model 1	1.89[b]	1.78–2.01	1.46[b]	1.33–1.61
	Model 2	1.72[b]	1.61–1.83	1.33[b]	1.22–1.46
	Model 3	1.69[b]	1.58–1.80	1.31[b]	1.20–1.44
	Model 4	1.69[b]	1.58–1.80	1.32[b]	1.20–1.44
Less vigorous-intensity activity than before pandemic (less activity vs. similar/more activity)	Model 1	1.37[b]	1.32–1.42	1.20[b]	1.14–1.26
	Model 2	1.34[b]	1.29–1.40	1.18[b]	1.12–1.24
	Model 3	1.36[b]	1.30–1.41	1.18[b]	1.13–1.24
	Model 4	1.36[b]	1.30–1.41	1.18[b]	1.13–1.24

[a] Model 1: crude model without adjustment for covariates; Model 2: adjusted for age, sex, educational level, country of birth, and household composition; Model 3: additionally adjusted for occupation, occupational class, and employment contract; Model 4: additionally adjusted for general health and testing positive for COVID-19; Model 5: additionally adjusted for moderate-to-vigorous-intensity activity before the pandemic.

[b] p<0.05.

Table S4. Effect estimates of all adjustment models[a] of the longitudinal associations between work situation and sedentary behavior (n=18,379). Reference group=location workers. [CI=confidence interval; OR=odds ratio].

		**Home workers**		**Hybrid workers**	
Sedentary behavior outcome measures	*Model*	*OR*	*95% CI*	*OR*	*95% CI*
Current sedentary behavior on workdays during the pandemic (sitting ≥8 hours vs. <8 per day)	Model 1	2.73[b]	2.61–2.87	2.16[b]	2.03–2.31
	Model 2	2.48[b]	2.36–2.61	1.99[b]	1.87–2.13
	Model 3	2.10[b]	2.00–2.22	1.82[b]	1.70–1.95
	Model 4	2.10[b]	1.99–2.22	1.81[b]	1.69–1.94
	Model 5	1.94[b]	1.83–2.06	1.73[b]	1.59–1.88
More sedentary on workdays during the pandemic than before the pandemic (more sitting vs. similar/less sitting)	Model 1	1.72[b]	1.60–1.85	1.66[b]	1.50–1.84
	Model 2	1.62[b]	1.50–1.75	1.58[b]	1.43–1.75
	Model 3	1.52[b]	1.40–1.64	1.52[b]	1.37–1.68
	Model 4	1.51[b]	1.39–1.64	1.51[b]	1.36–1.68
Less sedentary on workdays during the pandemic than before the pandemic (less sitting vs. similar/more sitting)	Model 1	1.24[b]	1.15–1.35	1.29[b]	1.15–1.44
	Model 2	1.14[b]	1.05–1.24	1.20[b]	1.08–1.34
	Model 3	0.95	0.87–1.04	1.06	0.95–1.19
	Model 4	0.95	0.87–1.03	1.06	0.95–1.18
Current sedentary behavior on weekend days during the pandemic (sitting ≥8 hours vs. <8 hours per day)	Model 1	1.44[b]	1.36–1.53	1.38[b]	1.28–1.50
	Model 2	1.46[b]	1.37–1.55	1.42[b]	1.30–1.54
	Model 3	1.39[b]	1.30–1.49	1.37[b]	1.26–1.49
	Model 4	1.38[b]	1.30–1.48	1.37[b]	1.25–1.49
	Model 5	1.32[b]	1.23–1.41	1.36[b]	1.23–1.50
More sedentary on weekend days during the pandemic than before the pandemic (more sitting vs. similar/less sitting)	Model 1	1.21[b]	1.12–1.30	1.39[b]	1.26–1.54
	Model 2	1.23[b]	1.14–1.33	1.43[b]	1.29–1.59
	Model 3	1.15[b]	1.06–1.25	1.37[b]	1.23–1.53
	Model 4	1.14[b]	1.05–1.24	1.36[b]	1.22–1.52
Less sedentary on weekend days during the pandemic than before the pandemic (less sitting vs. similar/more sitting)	Model 1	0.78[b]	0.69–0.87	1.06	0.92–1.21
	Model 2	0.75[b]	0.67–0.85	1.04	0.91–1.20
	Model 3	0.72[b]	0.64–0.81	1.01	0.88–1.16
	Model 4	0.72[b]	0.64–0.81	1.01	0.88–1.16

[a] Model 1: crude model without adjustment for covariates; Model 2: adjusted for age, sex, educational level, country of birth, and household composition; Model 3: additionally adjusted for occupation, occupational class, and employment contract; Model 4: additionally adjusted for general health and testing positive for COVID-19; Model 5: additionally adjusted for sedentary behavior before the pandemic.

[b] $p<0.05$.

Table S5. Effect estimates[a] of the longitudinal associations between work situation and physical activity among white-collar workers (n=28,677). Reference group=location workers. [CI=confidence interval; OR=odds ratio].

	**Home workers**		**Hybrid workers**	
Physical activity outcome measures	*OR*	*95% CI*	*OR*	*95% CI*
Current moderate-to-vigorous-intensity activity during pandemic (≥150 minutes vs. <150 minutes per week)	0.93[b]	0.90–0.96	1.03	0.98–1.08
More moderate-to-vigorous-intensity activity than before pandemic (more activity vs. similar/less activity)	0.92[b]	0.88–0.95	1.05	0.99–1.10
Less moderate-to-vigorous-intensity activity than before pandemic (less activity vs. similar/more activity)	1.10[b]	1.05–1.15	1.04	0.98–1.11
More vigorous-intensity activity than before pandemic (more activity vs. similar/less activity)	1.67[b]	1.56–1.79	1.32[b]	1.20–1.45
Less vigorous-intensity activity than before pandemic (less activity vs. similar/more activity)	1.36[b]	1.30–1.42	1.18[b]	1.13–1.24

[a] Adjusted for age, sex, educational level, country of birth, household composition, occupation, occupational class, employment contract, general health, testing positive for COVID-19. The fully adjusted model for the outcome measure current moderate-to-vigorous-intensity activity during the pandemic is additionally adjusted for moderate-to-vigorous-intensity activity before the pandemic.
[b] $p<0.05$.

Table S6. Effect estimates[a] of the longitudinal associations between work situation and sedentary behavior among white-collar workers (n=16,010). Reference group=location workers. [CI=confidence interval; OR=odds ratio].

	**Home workers**		**Hybrid workers**	
Sedentary behavior outcome measures	*OR*	*95% CI*	*OR*	*95% CI*
Current sedentary behavior on workdays during the pandemic (sitting ≥8 hours vs. <8 hours per day)	1.92[b]	1.80–2.03	1.70[b]	1.56–1.84
More sedentary on workdays during the pandemic than before the pandemic (more sitting vs. similar/less sitting)	1.49[b]	1.37–1.61	1.47[b]	1.32–1.63
Less sedentary on workdays during the pandemic than before the pandemic (less sitting vs. similar/more sitting)	0.96	0.88–1.05	1.07	0.95–1.19
Current sedentary behavior on weekend days during the pandemic (sitting ≥8 hours vs. <8 hours per day)	1.31[b]	1.22–1.41	1.36[b]	1.23–1.50
More sedentary on weekend days during the pandemic than before the pandemic (more sitting vs. similar/less sitting)	1.13[b]	1.04–1.23	1.37[b]	1.22–1.53
Less sedentary on weekend days during the pandemic than before the pandemic (less sitting vs. similar/more sitting)	0.73[b]	0.64–0.82	1.02	0.89–1.18

[a] Adjusted for age, sex, educational level, country of birth, household composition, occupation, occupational class, employment contract, general health, testing positive for COVID-19. The fully adjusted model for the outcome measures current sedentary behavior during the pandemic are additionally adjusted for sedentary behavior before the pandemic.
[b] $p<0.05$.

Table S7. Effect estimates[a] of the longitudinal associations between work situation and physical activity among workers with a high educational level (n=15,161). Reference group=location workers. [CI=confidence interval; OR=odds ratio].

	**Home workers**		**Hybrid workers**	
Physical activity outcome measures	*OR*	*95% CI*	*OR*	*95% CI*
Current moderate-to-vigorous-intensity activity during pandemic (≥150 minutes vs. <150 minutes per week)	0.90[b]	0.87–0.94	0.99	0.94–1.05
More moderate-to-vigorous-intensity activity than before pandemic (more activity vs. similar/less activity)	0.89[b]	0.85–0.93	0.99	0.93–1.05
Less moderate-to-vigorous-intensity activity than before pandemic (less activity vs. similar/more activity)	1.12[b]	1.06–1.19	1.05	0.97–1.13
More vigorous-intensity activity than before pandemic (more activity vs. similar/less activity)	1.57[b]	1.44–1.71	1.23[b]	1.10–1.38
Less vigorous-intensity activity than before pandemic (less activity vs. similar/more activity)	1.36[b]	1.29–1.43	1.19[b]	1.12–1.27

[a] Adjusted for age, sex, country of birth, household composition, occupation, occupational class, employment contract, general health, testing positive for COVID-19. The fully adjusted model for the outcome measure current moderate-to-vigorous-intensity activity during the pandemic is additionally adjusted for moderate-to-vigorous-intensity activity before the pandemic.
[b] p<0.05.

Table S8. Effect estimates[a] of the longitudinal associations between work situation and sedentary behavior among workers with a high educational level (n=8536). Reference group=location workers. [CI=confidence interval; OR=odds ratio].

	**Home workers**		**Hybrid workers**	
Sedentary behavior outcome measures	*OR*	*95% CI*	*OR*	*95% CI*
Current sedentary behavior on workdays during the pandemic (sitting ≥8 hours vs. <8 hours per day)	1.94[b]	1.79–2.09	1.71[b]	1.54–1.89
More sedentary on workdays during the pandemic than before the pandemic (more sitting vs. similar/less sitting)	1.51[b]	1.36–1.67	1.45[b]	1.27–1.66
Less sedentary on workdays during the pandemic than before the pandemic (less sitting vs. similar/more sitting)	0.86[b]	0.77–0.96	0.97	0.85–1.12
Current sedentary behavior on weekend days during the pandemic (sitting ≥8 hours vs. <8 hours per day)	1.31[b]	1.19–1.45	1.36[b]	1.19–1.54
More sedentary on weekend days during the pandemic than before the pandemic (more sitting vs. similar/less sitting)	1.12[b]	1.00–1.25	1.34[b]	1.16–1.54
Less sedentary on weekend days during the pandemic than before the pandemic (less sitting vs. similar/more sitting)	0.69[b]	0.59–0.80	0.95	0.80–1.13

[a] Adjusted for age, sex, country of birth, household composition, occupation, occupational class, employment contract, general health, testing positive for COVID-19. The fully adjusted model for the outcome measures current sedentary behavior during the pandemic are additionally adjusted for sedentary behavior before the pandemic.
[b] p<0.05.
